# Vertical jump without arm swing and running biomechanics in preschool children (3–6 years): associations and developmental insights

**DOI:** 10.3389/fspor.2026.1819016

**Published:** 2026-05-14

**Authors:** Jera Gregorc, Sanja Ljubičić, Vilko Petrić

**Affiliations:** 1 Faculty of Education, University of Ljubljana, Ljubljana, Slovenia; 2 Faculty of Teacher Education, University of Rijeka, Rijeka, Croatia

**Keywords:** fundamental motor skills, locomotor development, preschool children, reactive strength index, running biomechanics, vertical jump

## Abstract

This study examined associations between vertical jump without arm swing and running biomechanics in 405 preschool children aged 3–6 years (53.5% girls) from kindergartens in Slovenia and Croatia. Vertical jump (CMJ) and 10-metre sprint performances were assessed using the OptoJump system, which captured temporal and spatial parameters, from which additional performance indices were derived. No significant sex differences were observed in jumping, while boys demonstrated higher running speed, shorter stride and swing times, and shorter ground contact phases. Moderate correlations were found between jump height, flight time, reactive strength index, and running speed, step length, and foot-ground contact duration, indicating shared biomechanical and functional mechanisms between jumping and running already present in early childhood. Individual variability was pronounced, highlighting the importance of personalized movement activities over sex-based differentiation. The findings suggest that activities targeting jumping mechanics may simultaneously support running efficiency, contributing to integrated locomotor skill development in preschool children. Limitations include the cross-sectional design and partial sprint measurement.

## 1 Introduction

Fundamental movement skills (FMS) are the building blocks for acquiring later and more complex motor skills (1, 2). They primarily develop during early childhood, typically between 2 and 8 years of age, as part of normal motor development (3). FMS are commonly classified into three main categories: (1) locomotor skills, which involve body displacement (e.g., walking, running, jumping); (2) object control skills, such as throwing and catching; and (3) stability skills, including balance and trunk stability (4).

This study examines locomotor skills, specifically running and jumping. Both movements are considered fundamental forms of locomotion and share certain biomechanical characteristics, most notably the presence of a flight phase (5). The flight phase is defined as a transient loss of contact with the ground. Despite this similarity, running and jumping differ substantially in movement organization, functional purpose, and task demands. Differences in movement organization are particularly evident in the temporal and spatial dimensions. Running is longer in duration than jumping and represents a cyclical movement performed in the horizontal direction (6), whereas jumping is shorter in duration and constitutes a discrete movement primarily executed in the vertical direction (7). Functionally, running aims to achieve displacement through the environment, whereas jumping primarily involves overcoming gravitational forces. The motor demands of running and jumping also differ: running relies on rapid cyclical alternation between ground contact and propulsion, efficient use of the elastic properties of the muscle–tendon system, and maintenance of dynamic balance between the flight and support phases. In contrast, jumping requires a single, well-coordinated production of explosive force, with a more pronounced contribution from the trunk and lower extremities to achieve maximal vertical force output (8).

In preschool children, the vertical jump without arm swing is a developmentally appropriate task that reduces coordinative complexity and minimizes the influence of upper-limb contribution (9, 10). Excluding arm swing allows a more direct assessment of lower-limb neuromuscular function, trunk stabilization, and stretch-shortening cycle behaviour, which are still developing in early childhood (7, 9–11). As inter-limb coordination and the timing of arm-leg coupling are not yet fully established in children aged 3–6 years (10), the no-arm-swing condition provides a more controlled framework for examining fundamental force–time characteristics of jumping.

Although the relationships between running and jumping have been well documented in older children and athletes (5, 12), research focusing on the preschool period remains limited. Bertozzi et al. (13) reported that associations between running and jumping are already present in preschool-aged children; however, their findings were based on a relatively small sample of 29 children aged 4–5 years. In addition, their study examined the relationship between standing long jump performance and a 10 m sprint.

The scarcity of studies examining the relationship between running and jumping in preschool children may be because both skills are still undergoing significant development during this period. This development is not necessarily linear or synchronous, but depends on motor experience, the level of motor competence, and the quality of the environment in which the child operates (14). Consequently, a child may display different developmental levels across various locomotor skills simultaneously, further supporting the investigation of relationships between individual skills. Chen et al. (15) reported that children with higher overall motor competence tend to perform better in both locomotor skills and tasks requiring force and speed production, suggesting that a functional link between running and jumping already exists in the preschool period.

In addition to age, sex is an important factor in the development of fundamental locomotor skills. Research on preschool-aged children often reports small or inconsistent sex differences in basic motor tasks (16–18). However, sex-related differences may first appear in movement organization, temporal parameters, and efficiency of force production before becoming evident in absolute performance outcomes (18). Therefore, examining the relationships between individual locomotor skills separately by sex is methodologically justified, as this approach allows a more nuanced understanding of developmental variability in locomotor patterns during the preschool years.

Investigating the relationship between running and jumping in preschool children is important for advancing understanding of the structure of motor development, as it provides insight into whether these locomotor skills are underpinned by shared motor mechanisms or develop relatively independently. Understanding these relationships also has substantial practical value, as it can inform the design of movement activities in early childhood education, facilitate early identification of motor difficulties, and support the development of interventions that promote multiple locomotor skills simultaneously, thereby fostering holistic motor development.

Specifically, the study examines associations between vertical jump temporal parameters (contact time, flight time) and derived performance indices (jump height, reactive strength index, and verticality) and key running biomechanical outcomes related to speed, step length, and ground contact characteristics.

Based on the outlined theoretical and empirical framework, this study aimed to determine whether statistically significant positive associations exist between selected running and vertical jumping parameters in preschool children, and whether the patterns of these associations differ by sex, potentially indicating shared motor and functional demands underlying both locomotor skills.

### 1.2 Sample

The study included 405 children (mean ± SD: age 4.9 ± 1.1 years; height 111.2 ± 9.3 cm; weight 20.0 ± 4.2 kg; 53.5% girls). Participants were recruited from kindergartens in Koper (Slovenia), Ljubljana (Slovenia), Rijeka (Croatia), and Zagreb (Croatia).

Inclusion criteria were children aged 3–6 years with typical development and without diagnosed locomotor, neurological, or cognitive disorders. Ethical approval was obtained from the Ethics Committee of the Faculty of Teacher Education, University of Rijeka (Approval No. uniri-mladi-drustv-23-37: KLASA: 641-01/24-01/03; URBROJ: 2170-1-38-10-24-2; uniri-iskusni-drustv-23-201: KLASA: 641-01/24-01/03; URBROJ: 2170-1-38-10-24-1).

Parents or legal guardians were informed of the aims, benefits, and potential risks of the study and provided written informed consent for their child's participation. The study was conducted in accordance with national regulations and the Declaration of Helsinki (World Medical Association, 2013). Data collection took place between May and November 2024.

### 1.3 Measurement protocol

Before testing, participants completed a standardised preparation protocol consisting of a warm-up and preparatory exercises lasting up to 15 min. Jumping and running performances were assessed using the OptoJump system (Microgate, Bolzano, Italy), a reliable and valid photoelectric system comprising parallel bars positioned 1 m apart and connected to a computer with dedicated software (version 1.13.24.0) for automated data acquisition. The OptoJump system is a photoelectric measurement system that directly records temporal parameters, specifically ground contact time and flight time. Based on these primary measurements, additional variables such as jump height, reactive strength index (RSI), and power were calculated. These variables should therefore be interpreted as derived performance indices rather than direct measures of force production.

Each task was explained and demonstrated to the participants. Children performed one familiarisation trial followed by three recorded trials, with the best attempt retained for further analysis. Testing was conducted indoors between 09:00 and 12:00 to ensure standardised conditions. Assessments were administered by kinesiologists experienced in paediatric motor development. All participants performed the tasks wearing sports footwear.

### 1.4 Jumping performance

Vertical jump performance was assessed using the countermovement jump (CMJ) without arm swing. This protocol was selected to reduce coordinative complexity and to isolate lower-limb neuromuscular function by minimising upper-limb contribution. Flight time was measured with an accuracy of 1/1000 s using the OptoJump system. Jump height was calculated using the equation: 9.81 × (flight time)^2^/8 (19).

Children were instructed to start from an upright standing position with the trunk fully extended, knees extended, and feet approximately shoulder-width apart. The arms were placed on the hips and maintained in this position throughout the movement to prevent arm swing contribution.

From the initial standing posture, participants performed a rapid downward movement to approximately 90° of knee flexion, immediately followed by a maximal vertical jump. They were instructed to jump “as high as possible” while keeping their hands on their hips and avoiding preparatory steps or additional countermovements.

Participants were required to maintain an upright trunk without excessive forward lean, keep both feet in contact with the ground during the eccentric phase, take off and land with both feet simultaneously. Trials were repeated if arm movement, asynchronous take-off, or loss of balance occurred.

The software automatically generated data on contact time (s) and flight time (s) as primary measurements. Additional variables, including jump height (cm), relative power (W/kg), cadence (steps/s), and verticality, were calculated based on these temporal parameters. The reactive strength index (RSI) was calculated as jump height divided by ground contact time and is interpreted as a derived performance index reflecting the efficiency of the stretch–shortening cycle.

Previous research in early school-aged children has demonstrated high reliability (ICC = 0.92–0.97) and very strong concurrent validity (r = 0.97–0.98) of CMJ measurements compared to other platforms (20). While direct evidence in preschool populations is limited, these findings support the use of simplified CMJ protocols as a reliable approach for assessing jumping performance in young children.

### 1.5 Running performance

Running performance was assessed using a 10-metre sprint test. The OptoJump system has demonstrated high validity and reliability, with excellent agreement for kinematic parameters (ICC = 0.96–0.99; mean bias = 0.4–2.7%) and high accuracy in assessing step and temporal variables (21).

Testing was conducted on a flat, unobstructed surface with clearly marked start and finish lines. Additional space beyond the finish line ensured safe deceleration. To minimise pacing or deceleration effects, 2-metre OptoJump bars were positioned in the central section of the 10-metre sprint. Therefore, the recorded steps primarily reflect mid-run running mechanics rather than the initial acceleration phase.

Participants started from a standing position and were allowed to self-select their preferred lead leg (22). They were instructed to position their toes at the starting line and sprint as fast as possible through the finish line. One assessor walked alongside the running lane to ensure safety.

The fastest sprint (0–10 m), defined as the trial with the shortest ground contact time, was retained for further analysis. The software automatically generated data on contact time (s, %), flight time (s, %), swing phase (s), step time (s), step height (cm), velocity (m/s), acceleration (m/s^2^), step length (cm), stride length (cm), cadence (steps/s; steps/min), contact phase (s, %), full foot support phase (s, %), and propulsion phase (s, %).

### 1.6 Data analysis

Data normality was assessed using the Kolmogorov–Smirnov (K–S) test, supplemented by visual inspection of data distribution. A significant D value indicated deviation from a normal (Gaussian) distribution, and such variables were classified as non-normally distributed. Descriptive statistics are presented as mean and standard deviation (SD) for continuous variables, and as frequency (n) and percentage (%) for categorical variables. Sex differences were analyzed using independent samples Student's t-tests for continuous variables and Pearson's chi-square test for categorical variables.

Associations between jumping and running performance variables were examined using Pearson's and Spearman's correlation coefficients (r) depending on data distribution. Pearson's correlation was applied when both variables were normally distributed without substantial skewness or kurtosis, while Spearman's correlation was used when these assumptions were not met. Ninety-five percent confidence intervals (95% CI) were calculated for all correlation coefficients. Correlation magnitudes were interpreted as small (r = 0.1–0.3), moderate (r = 0.3–0.5), large (r = 0.5–0.7), very large (r = 0.7–0.9), and nearly perfect (r = 0.9–1.0) (32). The best trial was retained for analysis to reflect maximal performance capacity, which is commonly applied in pediatric motor testing. In young children, averaging across trials may be influenced by inconsistent effort, attentional fluctuations, and rapid fatigue. Therefore, the best trial approach is considered more representative of a child's actual motor potential. However, this approach may overestimate performance and potentially inflate associations, which should be considered when interpreting the results.

To control for type I error due to multiple comparisons, a Bonferroni correction was applied. The adjusted significance threshold was set at *p* < 0.007.

Given the observed sex-related differences in performance outcomes, correlation analyses were conducted for the total sample and separately for boys and girls. All statistical analyses were performed using SPSS version 27.0 (IBM Corp., Armonk, NY, USA). Statistical significance was set *a priori* at *p* < 0.05; however, for correlation analyses, a Bonferroni-adjusted threshold of *p* < 0.007 was applied.

## 2 Results

Descriptive statistics of jumping and running performance are presented in Table 1. Sex-related differences were examined to determine potential performance disparities.

**Table 1:** Jumping and running performance outcomes in pre-school boys and girls; data are presented as mean ± SD or median (IQR).

Study variables	Total sample (*n* = 405)	Boys (*n* = 186)	Girls (*n* = 219)	*P* – value
Jumping performance				
Contact time (s)	1.38 ± 0.99	1.41 ± 0.96	1.36 ± 1.01	0.599
Flight time (s)	0.30 ± 0.05	0.30 ± 0.05	0.30 ± 0.05	0.988
Height (cm)	11.3 ± 3.7	11.3 ± 3.8	11.2 ± 3.6	0.890
Power (W/kg)	9.5 ± 3.3	9.4 ± 3.2	9.5 ± 3.5	0.837
Pace (steps/s)	0.69 (0.53 - 1.11)	0.74 (0.52 - 1.30)	0.67 (0.53 - 1.02)	0.231
RSI (m/s)	0.09 (0.06 - 0.18)	0.10 (0.05 - 0.19)	0.09 (0.06 - 0.17)	0.767
Verticality	2.38 (1.29 - 4.58)	2.50 (1.27 - 5.01)	2.29 (1.28 - 4.13)	0.465
Running performance				
Contact time (s)	0.18 ± 0.03	0.18 ± 0.03	0.18 ± 0.02	0.088
Flight time (s)	0.07 ± 0.05	0.07 ± 0.03	0.07 ± 0.06	0.067
Swing phase (s)	0.30 ± 0.04	0.29 ± 0.04	0.31 ± 0.04	<0.001
Stride time (s)	0.48 ± 0.05	0.46 ± 0.05	0.49 ± 0.05	<0.001
Speed (m/s)	3.29 ± 0.51	3.37 ± 0.50	3.22 ± 0.51	0.005
Acceleration (m/s^2^)	0.36 ± 0.84	0.45 ± 0.49	0.29 ± 0.72	0.134
Step length (cm)	80.90 ± 13.44	79.90 ± 13.50	80.40 ± 13.50	0.732
Stride length (cm)	136.33 ± 21.74	135.90 ± 21.90	136.60 ± 21.80	0.752
Pace (steps/s)	4.13 ± 0.50	4.26 ± 0.55	4.02 ± 0.44	<0.001
Contact phase (s)	0.017 ± 0.010	0.015 ± 0.009	0.019 ± 0.010	<0.001
Foot flat (s)	0.082 ± 0.020	0.079 ± 0.021	0.085 ± 0.020	0.004
Propulsive phase (s)	0.080 ± 0.020	0.082 ± 0.021	0.079 ± 0.019	0.109

*P* < 0.05.

As shown in Table 1, no significant sex-related differences were observed in jumping performance variables (*p* > 0.05). In contrast, significant differences were found in several running parameters, with boys demonstrating shorter swing phase and stride time, higher running speed and pace, and shorter contact and foot flat phases compared to girls (*p* < 0.05). No differences were observed in running contact time, flight time, acceleration, step length, stride length, or propulsive phase (*p* > 0.05).

Correlations between jumping and running performance variables are presented in Table 2.

**Table 2:** Correlations between jumping and running performance variables values are presented as correlation coefficients (r). Significant correlations at *p* < 0.007 (Bonferroni-corrected).

Study variables	Jumping performance without arm swing						
	Contact time (s)	Flight time (s)	Height (cm)	Power (W/kg)	Pace (steps/s)	RSI (m/s)	Verticality
Running performance							
Contact time (s)	−0.03	0.04	0.04	0.03	−0.01	0.01	0.01
Flight time (s)	−0.04	0.05	0.05	0.04	−0.01	0.02	0.01
Swing phase (s)	0.04	0.09	0.09	0.06	−0.13	−0.01	−0.20*
Stride time (s)	0.09	−0.02	−0.02	−0.04	−0.19*	−0.09	−0.24*
Speed (m/s)	−0.29*	0.57*	0.57*	0.50*	0.18*	0.37*	0.15*
Acceleration (m/s^2^)	−0.11	0.09	0.10	0.08	0.21*	0.07	0.03
Step (cm)	−0.21*	0.55*	0.55*	0.43*	0.07	0.28*	0.08
Stride (cm)	−0.20*	0.39*	0.37*	0.34*	0.10	0.24*	0.03
Pace (steps/s)	−0.08	−0.03	−0.02	0.04	0.16*	0.08	0.08
Contact phase (s)	0.03	−0.03	−0.04	−0.07	−0.09	−0.08	−0.13
Foot flat phase (s)	0.12	−0.39*	−0.39*	−0.26*	−0.03	−0.16*	−0.08
Propulsive phase (s)	0.002	0.19*	0.18*	0.07	−0.06	0.01	−0.02

* *p* < 0.007 (Bonferroni corrected)  .

Jumping variables (jump height, RSI, power) are derived from temporal measurements and should be interpreted as performance indices.

As shown in Table 2, in the total sample several significant correlations were observed between jumping and running performance variables. Longer jump contact time was negatively correlated with running speed, step length, and stride length (*p* < 0.007 after the Bonferroni correction). Flight time was positively correlated with running speed, step length, stride length, and propulsive phase, and negatively correlated with foot flat phase (*p* < 0.05). Jump height and power were positively correlated with running speed, step length, and stride length, and negatively correlated with foot flat phase (*p* < 0.05). Jumping pace was positively correlated with running acceleration and running pace and negatively correlated with stride time (*p* < 0.05). Reactive strength index (RSI) was positively correlated with running speed, step length, and stride length, and negatively correlated with foot flat phase (*p* < 0.05). Verticality was negatively correlated with swing phase, and stride time, and positively correlated with running speed (*p* < 0.007 after the Bonferroni correction).

## 3 Discussion

In this study, the findings are discussed in relation to shared biomechanical and functional mechanisms underlying jumping and running, the strength of the relationships between these two locomotor skills, and the developmental characteristics of the preschool period, which are reflected in pronounced movement variability. Particular attention is also given to similarities and differences between boys and girls.

The first key finding concerns the presence of common mechanisms linking jumping and running already in the preschool years. Parameters of the vertical jump without arm swing were associated with several running characteristics, including speed, step length, and the duration of the foot flat phase. This suggests that shared biomechanical and functional foundations of jumping and running are established early in development. Children who achieved greater jump height, longer flight time, or a higher reactive strength index (RSI) also ran faster, used longer steps, and demonstrated shorter foot-ground contact times, indicating more effective neuromuscular coordination (10, 23, 24). The moderate magnitude of the observed correlations likely reflects the limited ability of preschool children to consistently exploit elastic energy and rapidly transition between eccentric and concentric muscle actions. At this age, variability in muscle activation timing, co-contraction, and tendon stiffness constrains the functional coupling between jumping and running.

This interpretation is further supported by the temporal characteristics of ground contact. A shorter foot flat phase in children with better jumping performance likely reflects a quicker transition from force absorption to propulsion, a key feature of effective stretch–shortening cycle (SSC) utilization in both jumping and running (25, 26). In this context, a higher RSI—reflecting the capacity for a rapid transition from eccentric to concentric muscle action—appears to play an important role in supporting running efficiency (26).

The second finding relates to the strength of the associations between jumping and running. While the results clearly support the existence of shared biomechanical and functional mechanisms, the moderate magnitude of the correlations indicates that these mechanisms are still developing in preschool children. Such moderate associations likely reflect the immaturity of the neuromuscular system at this age (27). In younger children, neuromuscular control is characterized by slower muscle activation, greater co-activation of agonists and antagonists, and lower tendon stiffness, which together contribute to variable movement patterns and only moderate links between jumping and running performance (27, 28).

Examining the strength of these relationships provides insight into how different locomotor skills are functionally integrated and where developmental constraints remain. Compared with adult athletes, in whom relationships between jumping and running are stronger and more consistent (12, 29), the present findings suggest that these mechanisms are still emerging in preschool children. Thus, although common biomechanical foundations are already present, children are not yet able to fully automatize or optimally coordinate jumping and running.

The third important observation relates to the substantial variability in movement performance. Large standard deviations and interquartile ranges were observed across most jumping and running parameters. For example, jump height was 11.3 ± 3.7 cm, RSI was 0.09 (0.06–0.18) m/s, stride time was 0.48 ± 0.05 s, and the foot flat phase lasted 0.082 ± 0.020 s. This dispersion highlights considerable individual differences among children, reflecting differences in neuromuscular maturity, motor competence, and movement experience (14).

Importantly, such variability should not be interpreted as random noise but as a defining feature of motor development in the preschool period. Children actively explore different movement solutions and gradually refine muscle coordination and SSC utilization, adapting both jumping and running to their individual capabilities (13, 15). These processes are particularly evident in differences in foot flat duration and in the speed of transitions between force absorption and propulsion, suggesting that efficient neuromuscular strategies for coordinating the lower limbs and trunk are still under development. Although the CMJ is a complex task to perform for preschool children, due to a developmental phase in this specific age-related period, the ecological validity of this movement mimics everyday activities from hopping, landing, and pushing (taking off) phases. Indeed, previous research has shown that the usefulness of the CMJ in preschool children was rated as ‘OK’, meaning that the smallest worthwhile change that occurs within each measure is higher or equal than the typical error. Indeed, it is somewhat expected that the results in the CMJ outcome are highly correlated with coordinative issues, which can have an impact on jump performances (roughly 10%), as a result of the shoulder, elbow, hip, and ankle muscles working together. However, the test eliminates movement patterns of upper extremities and focuses mainly on concentric and eccentric phases of the jump (30, 31).

The fourth finding concerns sex-related differences in jumping and running performance. Absolute differences between boys and girls were small and often negligible [e.g., jump height: boys 11.3 ± 3.8 cm, girls 11.2 ± 3.6 cm; RSI: boys 0.10 [0.05–0.19] m/s, girls 0.09 [0.06–0.17] m/s; *p* > 0.05], even though some running parameters showed statistically significant differences (e.g., swing phase, stride time, speed, pace, foot flat phase). However, these differences were consistently smaller than within-sex variability, indicating that individual differences are more pronounced than sex differences at this age. The observed sex-specific patterns in correlations, despite minimal differences in absolute performance, may indicate differences in movement strategies rather than differences in physical capacity.

This is consistent with previous findings (16–18), which suggest that during the preschool period sex differences are expressed primarily in movement organization, temporal characteristics, and force production efficiency, while absolute motor outcomes remain largely comparable between boys and girls. These findings support an approach that prioritizes individual abilities and task demands over sex-based differentiation.

Given the number of correlations examined, findings should be interpreted with an emphasis on effect size and consistency of patterns rather than statistical significance alone. The strongest and most consistent associations were observed for parameters directly related to speed and ground contact mechanics. It should be noted that several jumping variables (e.g., jump height, RSI, and power) are mathematically derived from primary temporal measurements (flight time and contact time). As such, these variables are not independent and reflect overlapping aspects of performance. Therefore, the observed associations should be interpreted as relationships between derived performance indices rather than direct biomechanical measures of force production.

## 4 Conclusion

The present study demonstrates that shared biomechanical and functional mechanisms between jumping and running are already present in the preschool period. However, the moderate strength of the associations, together with pronounced movement variability, reflects ongoing neuromuscular development. The predominance of individual over sex-related differences underscores the importance of individualized approaches when planning movement activities for preschool children. From a practical perspective, the findings suggest that activities targeting jumping mechanics in early childhood may simultaneously support the development of efficient running patterns, highlighting the value of integrated locomotor tasks in preschool movement education.

## 5 Limitations

Several limitations should be acknowledged. First, the cross-sectional design limits the interpretation of the findings to associations and does not allow causal inferences. Second, the age range of the participants (3–6 years) restricts the generalization of the results to older children. Third, age-related heterogeneity within the preschool period may have influenced the strength of observed associations, as neuromuscular development progresses rapidly between 3 and 6 years of age. Fourth, only a short segment of the OptoJump system was available for biomechanical analysis, which limits the capture of the complete running pattern. A key limitation of the study is that running mechanics were assessed over a short segment located in the middle of the 10-metre sprint. Consequently, the data primarily reflect mid-run mechanics rather than the initial acceleration phase, which may limit the generalizability of findings to early sprint performance. Future studies should adopt a longitudinal design to track the development of stretch–shortening cycle function across different fundamental movement skills, which would provide deeper insight into locomotor development during early childhood.

## Data Availability

The datasets generated during the current study are available from the corresponding author, Vilko Petrić, upon reasonable request.
